# Interaction between MyRIP and the actin cytoskeleton regulates Weibel–Palade body trafficking and exocytosis

**DOI:** 10.1242/jcs.178285

**Published:** 2016-02-01

**Authors:** Ianina L. Conte, Nicola Hellen, Ruben Bierings, Gregory I. Mashanov, Jean-Baptiste Manneville, Nikolai I. Kiskin, Matthew J. Hannah, Justin E. Molloy, Tom Carter

**Affiliations:** 1 Cardiovascular and Cell Science Research Institute, St George's University, London SW17 0RE, UK; 2 The Francis Crick Institute, Mill Hill Laboratory, London NW7 1AA, UK; 3 UMR144 CNRS/Institute Curie, Paris 75248, France

**Keywords:** Actin, Exocytosis, MyRIP, Myosin Va, Weibel–Palade body

## Abstract

Weibel–Palade body (WPB)–actin interactions are essential for the trafficking and secretion of von Willebrand factor; however, the molecular basis for this interaction remains poorly defined. Myosin Va (MyoVa or MYO5A) is recruited to WPBs by a Rab27A–MyRIP complex and is thought to be the prime mediator of actin binding, but direct MyRIP–actin interactions can also occur. To evaluate the specific contribution of MyRIP–actin and MyRIP–MyoVa binding in WPB trafficking and Ca^2+^-driven exocytosis, we used EGFP–MyRIP point mutants with disrupted MyoVa and/or actin binding and high-speed live-cell fluorescence microscopy. We now show that the ability of MyRIP to restrict WPB movement depends upon its actin-binding rather than its MyoVa-binding properties. We also show that, although the role of MyRIP in Ca^2+^-driven exocytosis requires both MyoVa- and actin-binding potential, it is the latter that plays a dominant role. In view of these results and together with the analysis of actin disruption or stabilisation experiments, we propose that the role of MyRIP in regulating WPB trafficking and exocytosis is mediated largely through its interaction with actin rather than with MyoVa.

## Abstract (precis)

**Summary:** The role of MyRIP in restricting the movement and exocytosis of Weibel–Palade bodies in endothelial cells is mediated primarily through its actin- rather than myosin-Va-binding properties.

## INTRODUCTION

Weibel–Palade bodies (WPBs) are endothelial cell secretory granules containing the adhesive glycoprotein von Willebrand factor (VWF). VWF secretion is triggered by physico-mechanical and chemical signals that increase intracellular free Ca^2+^ ([Ca^2+^]_i_) or cAMP concentrations (Rondaij et al., 2006b). Mutations in VWF that result in a qualitative or quantitative defect in function cause a family of bleeding disorders collectively called von Willebrand disease (Sadler et al., 2006). Molecular defects affecting the trafficking or exocytosis of WPBs can also give rise to alterations in circulating VWF and cause bleeding disorders (van Loon et al., 2010; Zhu et al., 2014).

Trafficking and exocytosis of WPBs depend on the interplay between microtubule transport and actin cytoskeleton tethering (Manneville et al., 2003; Rojo Pulido et al., 2011; Rondaij et al., 2006a; Vischer et al., 2000). WPB movements on microtubules are reduced through interactions with actin (Manneville et al., 2003), which might help prevent untimely WPB exocytosis, allowing VWF multimerization to proceed to its biologically more potent high-molecular-mass form (Nightingale et al., 2009). However, the precise molecular basis for WPB–actin interactions remain poorly defined.

Specialised secretory cells express one or more of a subgroup of small GTPases called ‘secretory Rabs’. These Rabs localise to the secretory granule membrane and recruit soluble effector molecules to regulate granule interactions with the cytoskeleton and the plasma membrane during trafficking and exocytosis (Fukuda, 2008). WPBs recruit the secretory Rabs, Rab27A and Rab3A, Rab3B and Rab3D, and the effectors MyRIP, Munc13-4 (also known as UNC13D) and Slp4 isoform a (Slp4-a; also known as SYTL4-a) (Bierings et al., 2012; Nightingale et al., 2009; Zografou et al., 2012). The current model places the molecular motor myosin Va (MyoVa or MYO5A) as the primary link mediating WPB binding to actin, with MyoVa being recruited to the granule through the Rab27A-specific effector MyRIP (Rojo Pulido et al., 2011). However, it has been reported that MyRIP can also bind actin directly through a C-terminal actin-binding motif, similarly to the MyRIP-related protein melanophilin (Waselle et al., 2003). Direct MyRIP–actin interaction has been reported to play a role in regulating secretory granule trafficking and exocytosis in endocrine and neuroendocrine cells (Huet et al., 2012; Waselle et al., 2003); however, its contribution to WPB trafficking and exocytosis has not yet been explored. To address this, we used total internal reflection fluorescence microscopy (TIRFM) and single-particle tracking to analyse the effect of MyRIP mutants with disrupted MyoVa and/or actin-binding. We demonstrate that the role of MyRIP can be only partly accounted for by its recruitment of MyoVa, and that MyRIP–actin interactions play a prominent role for both trafficking and exocytosis.

## RESULTS

### Ca^2+^-driven WPB exocytosis after actin disruption or stabilisation

**Fig. 1.**
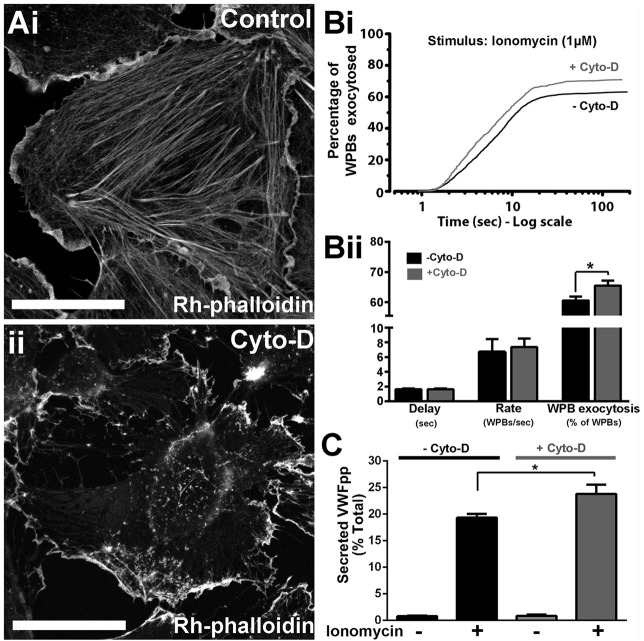
**The actin cytoskeleton limits Ca^2+^-driven WPB exocytosis.** (A) Rhodamine (Rh)–phalloidin labelling of HUVECs after a 20-min incubation with vehicle (0.1% DMSO) (i), or 1 µM cytochalasin D (Cyto-D) (ii). Scale bars: 10 µm. (Bi) Cumulative plots of the total number of WPB fusion events detected during ionomycin stimulation (1 µM, applied at *t*=0 s) of VWFpp–EGFP-expressing HUVECs pre-treated for 20 min with vehicle [0.1% DMSO, no cytochalasin D (−Cyto-D), black, *n*=954] or 1 µM Cyto-D (+Cyto-D; grey, *n*=934). Data are scaled to the mean fraction of fluorescent WPBs that underwent fusion in each condition (−Cyto-D, 61.1±2.6%, *n*=19 cells; +Cyto-D, 71.0±3.3%, *n*=23 cells; mean±s.e.m.). (Bii) Summary of the mean±s.e.m. delay, maximum rate and percentage of fluorescent WPBs exocytosed in control (black, *n*=19) and Cyto-D-treated (grey, *n*=23) cells. *P=0.013, *t*-test. (C) Ionomycin-stimulated secretion of endogenous VWFpp in control; −Cyto-D (black) or +Cyto-D (grey) cells. Plots show data pooled from three independent experiments performed in triplicate (mean±s.e.m., *n*=9 replicates). *Adjusted *P*=0.011, ANOVA, Tukey multiple comparisons test.

To directly assess the effect of actin disruption on the kinetics of Ca^2+^-driven WPB exocytosis, we used live-cell imaging of human umbilical vein endothelial cells (HUVECs) expressing the WPB-specific luminal marker EGFP-tagged VWF propolypeptide (VWFpp–EGFP) (Hannah et al., 2005), which does not directly interfere with interactions between WPBs and cytoskeletal components or plasma membrane during exocytosis. Actin was disrupted by cytochalasin D treatment (Fig. 1A) prior to stimulation with ionomycin, and WPB fusion events plotted cumulatively as the percentage of WPBs fused in each cell against time as previously described (Hewlett et al., 2011) (Fig. 1Bi). The effect of actin disruption was subtle: there was no significant change in the initial delay or the mean maximal rate of WPB fusion (Fig. 1Bii); however, Fig. 1Bi shows that, shortly after stimulation (1–3 s), the rate of WPB fusion in treated cells was slightly greater than in control cells. This initial increased rate might account for the small, but significant, increase in the overall number of fusion events observed in treated cells (Fig. 1Bi,ii), and was mirrored by a significant increase in stimulated, but not unstimulated (basal), VWFpp secretion (Fig. 1C). These results show that the net effect of actin cytoskeleton is to prevent Ca^2+^-driven WPB secretion.

**Fig. 2.**
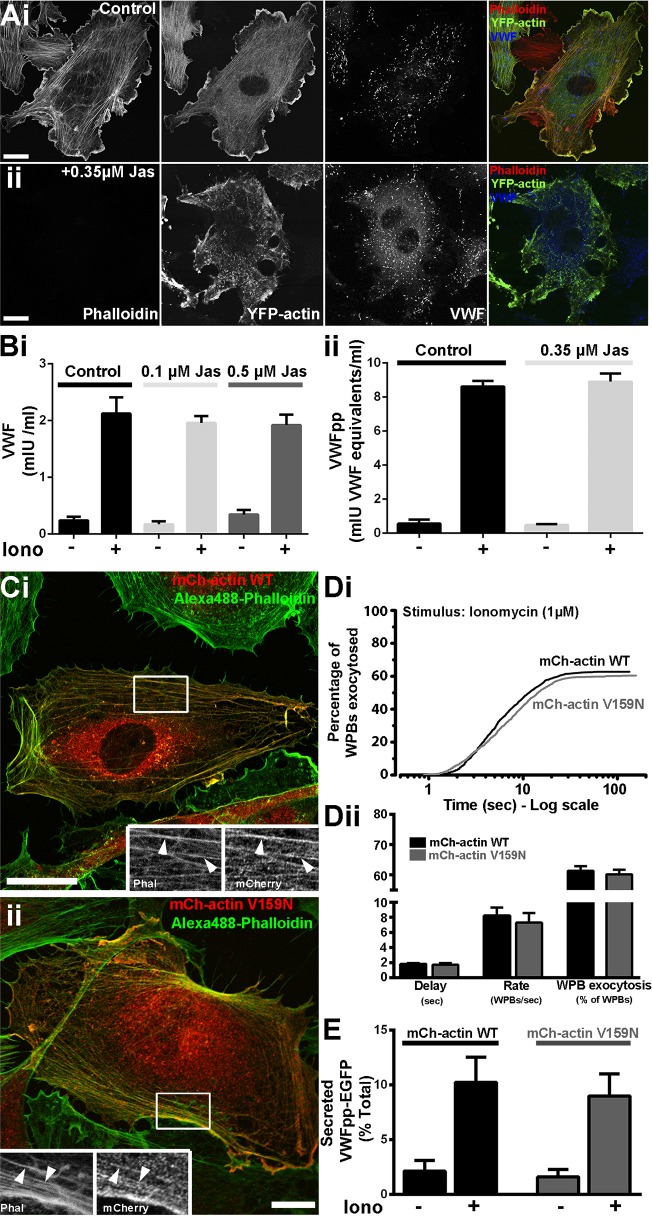
**Actin remodelling is not required for Ca^2+^-driven WPB exocytosis.** (A) Effect of jasplakinolide on actin cytoskeleton and cell morphology. Images show the effect of a 20-min incubation with vehicle (i) or 0.35 µM jasplakinolide (Jas, ii) on the actin cytoskeleton and cell morphology visualised by expression of YFP–actin (green in colour merged images). Competitive binding of jasplakinolide to actin is manifested by the displacement of Rhodamine–phalloidin staining (red in colour merged images). Scale bars: 10 µm. (B) Effect of a 20-min pre-treatment of HUVECs with jasplakinolide (at the indicated concentrations) on ionomycin-evoked (1 µM) VWF (i) or VWFpp (ii) secretion. Stimulation was for 10 min in the continued presence of vehicle or jasplakinolide. Experiments shown are representative of thre independent experiments performed in triplicate (mean±s.e.m.). (C) Images of single HUVECs 24 h after nucleofection with mCherry–actin WT (red; anti-RFP antibody) (i), or mCherry–actin V159N (red; anti-RFP antibody) (ii), and counterstained with Alexa-Fluor-488–phalloidin (green). Regions indicated by white boxes are shown as greyscale inserts. Arrowheads indicate colocalisation of RFP fluorescence with actin filaments. Scale bars: 10 µm. (Di) Cumulative plots of the total number of WPB fusion events detected during ionomycin stimulation (as in Fig. 1Bi) of HUVECs co-expressing VWFpp–EGFP and mCherry–actin WT (black, *n*=1038), or mCherry–actin V159N (grey, *n*=999). Data are scaled to the mean fraction of fluorescent WPBs that underwent fusion in each case (actin WT, 62.8±2.9%, *n*=21 cells; actin V159N, 60.4±3.0%, *n*=21 cells; mean±s.e.m.). (Dii) Summary of the delay, maximum rate and percentage of fluorescent WPBs exocytosed for cells expressing mCherry–actin WT (black; mean±s.e.m., *n*=21) or mCherry-actin V159N (grey; mean±s.e.m., *n*=21). (E) Ionomycin-stimulated secretion of VWFpp–EGFP in mCherry–actin WT (black) or mCherry–actin V159N (grey) co-expressing cells. Plots show data pooled from three independent experiments performed in triplicate (mean±s.e.m.).

To examine the role of actin dynamics in WPB exocytosis, we stabilized actin filaments using the cyclic peptide jasplakinolide. To determine an effective concentration of jasplakinolide, we monitored the displacement of Rhodamine–phalloidin fluorescence by this peptide, to confirm binding to F-actin, and used expressed YFP–actin to visualise the actin cytoskeleton and cell morphology. Partial or complete phalloidin displacement was seen at ≥100–350 nM jasplakinolide (20 min) (Fig. 2Ai,ii; Fig. S1); however, a profound disruption of the actin cytoskeleton and cell morphology was observed at concentrations ≥1 µM (Fig. S1). Based on this, we used 100–500 nM jasplakinolide, and found no inhibition of Ca^2+^-evoked VWF (Fig. 2Bi) or VWFpp (Fig. 2Bii) secretion. To examine this further, we expressed an mCherry-tagged actin V159N mutant that has previously been shown to stabilise F-actin (Posern et al., 2004). Both WT and V159N actins localised to stress fibres and cortical actin with no visible disruption to cell morphology (Fig. 2Ci,ii). Under these conditions, neither the kinetics or extent of WPB fusion (Fig. 2Di,ii), or VWFpp–EGFP secretion (Fig. 2E) were significantly altered, suggesting that acute actin remodelling is not required for regulating Ca^2+^-driven WPB exocytosis. Because jasplakinolide-mediated disruption of actin dynamics has been reported to perturb MyoVa-driven organelle transport (Semenova et al., 2008), the data in Fig. 2 suggest that the reported role of MyoVa in WPB exocytosis (Rojo Pulido et al., 2011) might reflect an effect on WPB docking or fusion rather than movement on the actin cytoskeleton.

### MyRIP–actin binding regulates WPB trafficking

**Fig. 3.**
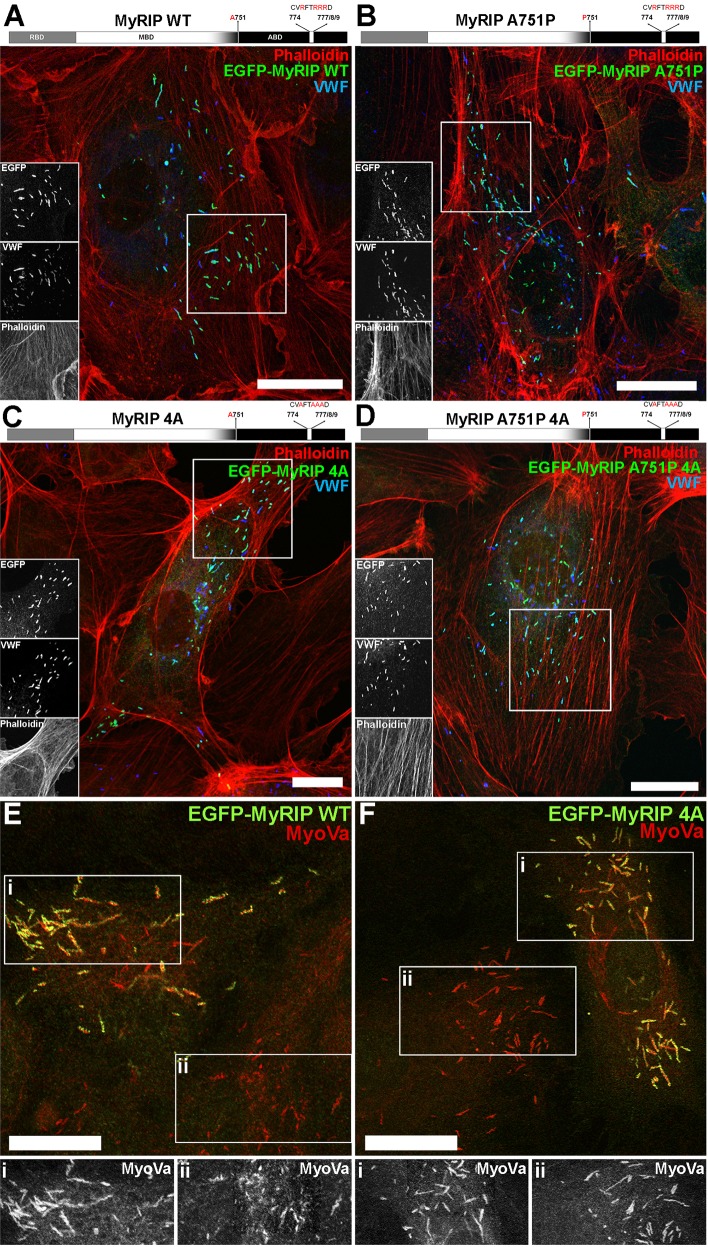
**Subcellular localisation of EGFP–MyRIP mutants in HUVECs – mutation of the actin-binding region does not perturb endogenous MyoVa recruitment to WPBs.** (A) Top, schematic structure of human MyRIP domains and position of the mutated amino acids. Below, confocal immunofluorescence image of a single HUVEC expressing EGFP–MyRIP WT and labelled with phalloidin (red) and specific antibodies against EGFP (green) and VWF (blue). Regions indicated by white boxes are shown as greyscale inserts. (B–D) As for A but for cells expressing EGFP–MyRIP A751P (B), EGFP–MyRIP 4A (C) and EGFP–MyRIP A751P 4A (D). (E,F) Confocal immunofluorescence images of single HUVECs expressing EGFP–MyRIP WT (E) or EGFP–MyRIP 4A (F), and labelled with specific antibodies to EGFP (green) and MyoVa (red). Greyscale images below show, on the same scale, endogenous MyoVa immunoreactivity from regions indicated in the MyRIP-expressing (i) and non-expressing (ii) cells. Scale bars: 10 µm.

In order to better understand the molecular interactions that regulate the motion of WPBs through the actin-rich layer, we focused our attention on MyRIP. MyRIP has an N-terminal domain that allows it to interact with Rab27A (Fukuda and Kuroda, 2002) and be recruited to WPBs (Nightingale et al., 2009) (Fig. 3A, top diagram). This domain is followed by a central myosin-binding domain and a C-terminal actin-binding domain. Although MyRIP can bind to both MyoVa and MyoVIIa (Fukuda and Kuroda, 2002), we did not detect endogenous MyoVIIa [by quantitative real-time PCR (qPCR), western blotting or immunocytochemistry] in the HUVEC cultures used here (data not shown) and therefore concentrated on the MyoVa- and actin-binding properties of MyRIP. To examine their individual contributions, we generated EGFP-tagged MyRIP mutants: MyRIP A751P, which has been previously shown to be defective in MyoVa recruitment and activation in melanosomes (Ramalho et al., 2009), MyRIP R774A, R777A, R778A, R779A (from now on referred to as MyRIP 4A) that is defective in actin binding (Waselle et al., 2003), and the combined mutant MyRIP A751P 4A, designed to disrupt both MyoVa and actin interactions (Fig. 3B–D). We chose this approach rather than the previously used C-terminal truncations to avoid confounding results due to the presence of specific residues crucial for both MyoVa- and actin-binding within this region (Ramalho et al., 2009). All MyRIP constructs retained the Rab27A-binding domain and were recruited to WPBs (Fig. 3A–D). To confirm that specific disruption of the actin-binding region within the C-terminal domain did not prevent MyoVa recruitment to the WPB, we examined the localisation of endogenous MyoVa in cells expressing EGFP–MyRIP A4 (Fig. 3E,F). No evidence for loss or depletion of endogenous MyoVa immunoreactivity from WPBs was seen.

**Fig. 4.**
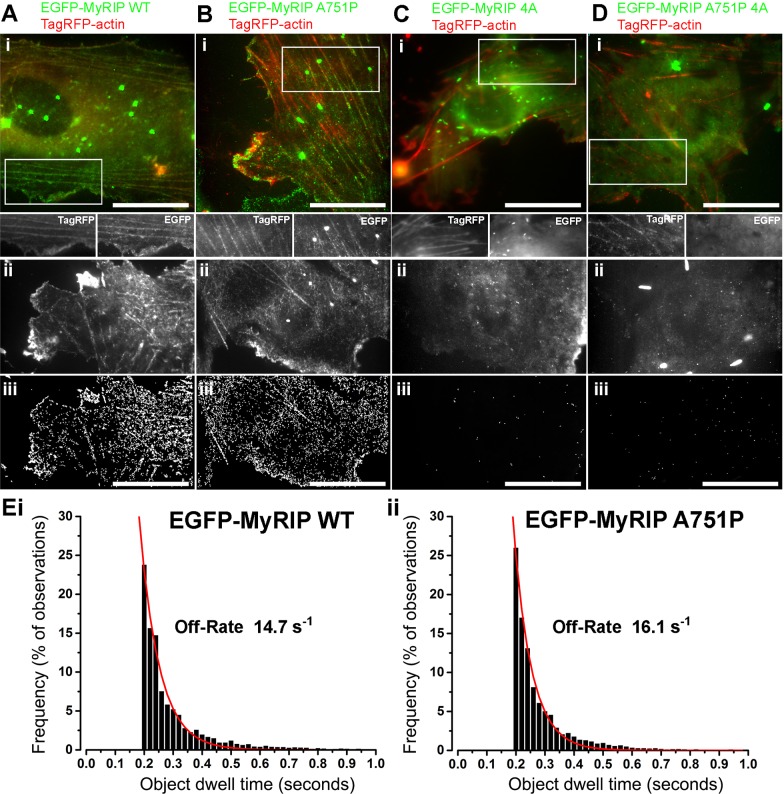
**Actin-binding properties of EGFP–MyRIP mutants.** (Ai–Di) Images from dual colour TIRF movies of live HUVECs co-expressing EGFP–MyRIP constructs (green) and TagRFP–actin (red) showing MyRIP WT (Ai) and MyRIP A751P (Bi), but not MyRIP 4A (Ci) or MyRIP A751P 4A (Di) decorating cortical actin and stress fibres. Images are averages of 134 to 222 consecutive frames acquired at 33 frames/s. Scale bars: 20 µm. Regions indicated by white boxes in Ai–Di are shown in greyscale below the colour merged images. Dual-colour video clips of a small sub-region of each cell are shown in Movies 1–4. (Aii–Dii) Representative examples of single-colour TIRF images of live HUVECs expressing MyRIP WT, MyRIP A751P, MyRIP 4A or MyRIP A751P 4A. Images are averages of 401–900 consecutive frames taken from videos acquired at 50 frames/s. To minimise false detection events during automatic single fluorophore detection and analysis, cells expressing EGFP–MyRIP mutants containing few or no WPBs were selected for recording. (Aiii–Diii) White tracks show the detected trajectories of single EGFP fluorophores for the cells in Aii–Dii (Aiii, 7731 detected objects; Biii, 6448 objects, Ciii, 54 objects; Dii, 89 objects). Scale bars: 20 µm. (E) Lifetime distributions for single fluorophore interaction of EGFP–MyRIP WT (i, *n*=12,703 objects from 16 cells) or EGFP–MyRIP A751P (ii, *n*=12,461 objects from 10 cells) and actin derived from experiments illustrated in Aii,iii or Bii,iii, respectively. Red lines show exponential fits (i, R=0.98; ii, R=0.99) to the distributions, and the off-rates determined from the fits are shown on the plots. Events with durations less than 200 ms were excluded to avoid short-lived ‘false’ events (see Materials and Methods).

To test whether the A751P mutation perturbed the actin binding properties of MyRIP we determined the off-rates for the MyRIP-actin interaction by high-resolution single molecule imaging and single molecule dwell time analysis in live cells. The data showed that cytosolic MyRIP WT and MyRIP A751P bind transiently to cortical actin and stress fibres (Fig. 4Ai–iii,Bi–iii; Movies 1, 2) with similar off-rates of 14.7 s^−1^ and 16.1 s^−1^, respectively (Fig. 4Ei,ii). In contrast, actin binding was abolished for MyRIP 4A and MyRIP A751P 4A (Fig. 4Ci–iii,Di–iii; Movies 3, 4). This argues that, in the free state, MyRIP binding to actin is independent of the ability of MyRIP to recruit MyoVa.

**Fig. 5.**
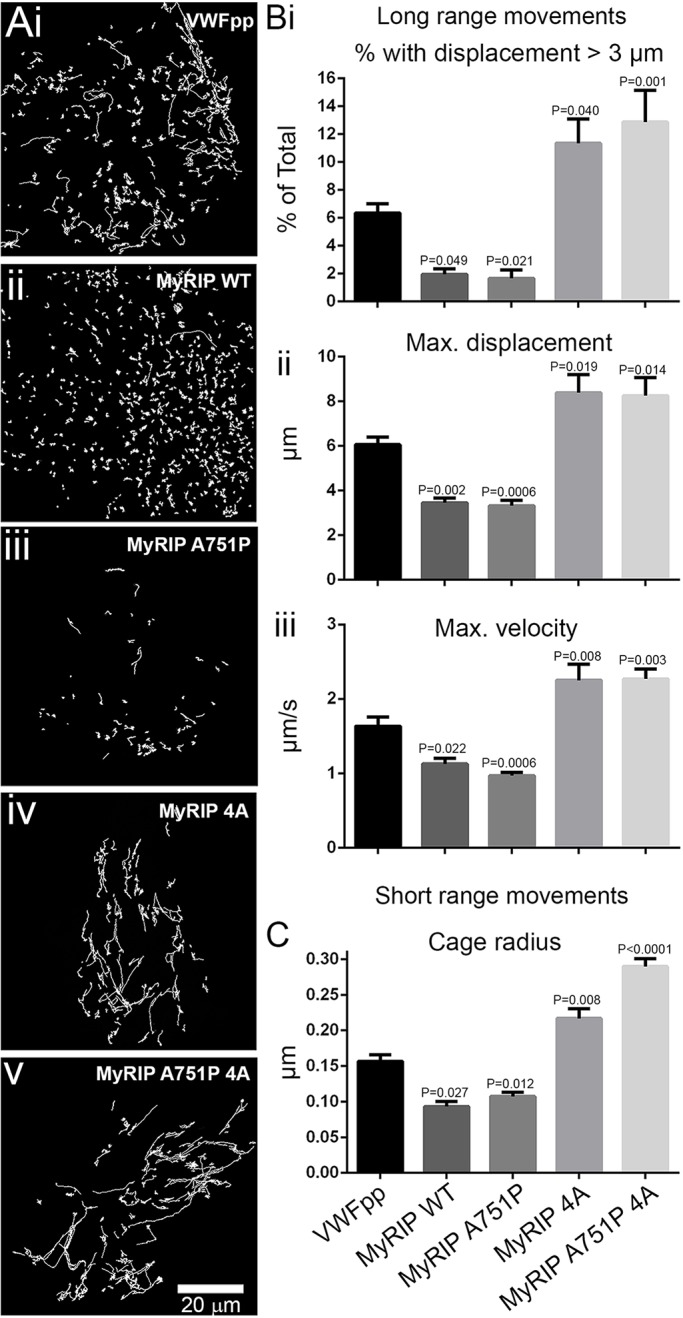
**MyRIP–actin interaction regulates WPB trafficking.** (A) Representative *x-y* trajectories of individual WPBs in single HUVECs expressing VWFpp–EGFP (i), EGFP–MyRIP WT (ii), EGFP–MyRIP A751P (iii), EGFP–MyRIP 4A (iv) or EGFP–MyRIP A751P 4A (v). Trajectories were determined here and elsewhere from TIRFM videos using the ASPT function of GMimPro software (see Materials and Methods). The number of cells imaged and trajectories detected were: VWFpp, *n*=15 cells, 3890 trajectories; MyRIP WT, *n*=11 cells, 2978 trajectories; MyRIP A751P, *n*=13 cells, 1278 trajectories; MyRIP 4A, *n*=8 cells, 1862 trajectories; MyRIP A751P 4A, *n*=11 cells, 1310 trajectories. (B,C) Mean±s.e.m. parameters determined from detected trajectories of long-range (Bi–iii) or short-range (C) movements of WPBs in HUVECs expressing EGFP fusion proteins of VWFpp, MyRIP WT and MyRIP mutants as indicated. Number of WPBs analysed for short-range movements were: VWFpp, *n*=277; MyRIP WT, *n*=86; MyRIP A751P, *n*=242; MyRIP 4A, *n*=250; MyRIP A751P 4A, *n*=387. *P* values are with respect to VWFpp–EGFP (one-way ANOVA using Tukey multiple comparisons test).

**Fig. 6.**
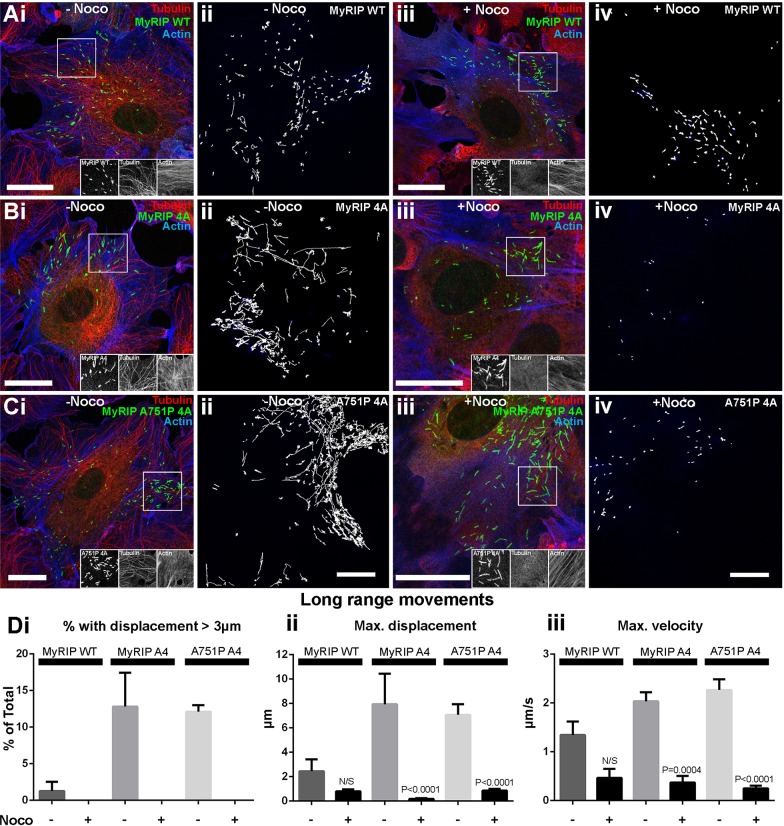
**Microtubule disruption abolishes long-range movements of WPBs carrying actin-binding-defective EGFP–MyRIP mutants.** (Ai–Ci) Representative confocal immunofluorescence images of individual HUVECs expressing EGFP fusion proteins of MyRIP WT (Ai), MyRIP 4A (Bi) or MyRIP A751P 4A (Ci) exposed to vehicle (0.1% DMSO, 20 min: −Noco) and labelled with specific antibodies to EGFP (green), α-tubulin (red) and actin (phalloidin; blue). Scale bars: 20 µm. (Aii–Cii) Representative examples of the *x-y* trajectories of individual WPBs in control (−Noco) HUVECs expressing EGFP fusion proteins of MyRIP WT (Aii), MyRIP 4A (Bii) or MyRIP A751P 4A (Cii). Number of cells imaged and trajectories detected were: MyRIP WT, *n*=7 cells, 1007 trajectories; MyRIP 4A, *n*=3 cells, 448 trajectories; MyRIP A751P 4A, *n*=8 cells, 1667 trajectories. Scale bar: 10 µm for Aii-Cii (shown in Cii). (Aiii–Ciii) Representative confocal immunofluorescence images of individual HUVECs expressing the same EGFP fusion proteins and stained as described in Ai–Ci, but after treatment with nocodozole (1 µM, 20 min: +Noco). Scale bars: 20 µm. (Aiv–Civ) Representative examples of the *x-y* trajectories of individual WPBs in +Noco treated HUVECs expressing EGFP fusion proteins of MyRIP WT (Aiv), MyRIP 4A (Biv) or MyRIP A751P 4A (Civ). Number of cells imaged and trajectories detected were: MyRIP WT, *n*=3 cells, 497 trajectories; MyRIP 4A, *n*=7 cells, 125 trajectories; MyRIP A751P 4A, *n*=10 cells, 392 trajectories. Scale bar: 10 µm for Aiv-Civ (shown in Civ). (Di–iii) Mean±s.e.m. values for the fraction of WPBs with movements greater than 3 µm in length (Di), maximum displacement from start of recording (Dii) and maximum velocity (Diii) determined from the detected trajectories of the WPBs in control (−Noco) or nocodozole (+Noco) treated cells expressing the MyRIP fusion proteins as indicated. Number of cells imaged and trajectories detected in −Noco and +Noco treated cells are as described above. *P* values were calculated with a one-way ANOVA using Tukey multiple comparisons test.

Analysis of the movements of WPBs overexpressing wild-type EGFP–MyRIP (EGFP–MyRIP WT) showed that there was a reduction in the fraction of WPBs exhibiting long-range movements, conceivably due to an increased interaction with F-actin that counteracts WPB motions on microtubules (Manneville et al., 2003). These WPBs also showed a reduction in their maximum displacement and velocity compared to those in cells expressing the WPB luminal marker VWFpp–EGFP (Fig. 5Ai,ii,B). Consistent with dwell time analysis, we found that WPBs carrying MyRIPA751P behaved identically to those carrying MyRIP WT (Fig. 5Aiii,B) suggesting that the brake that MyRIP exerts on the movement of WPBs is not mediated by MyoVa. Conversely, a higher proportion of WPBs carrying MyRIP 4A showed longer trajectories and faster movements (Fig. 5Aiv,B), and no additional increases were seen for WPBs carrying the combined mutant (Fig. 5Av,B). A similar phenotype was seen for VWFpp–EGFP-labelled WPBs after depletion of endogenous MyRIP by small interfering RNA (siRNA) (Fig. S2B), strongly indicating that MyRIP–actin interactions are the ones primarily responsible for the role of MyRIP in WPB trafficking. In addition, rapid long-range WPB movements in cells expressing MyRIP WT, MyRIP 4A or MyRIP A751P 4A were abolished by nocodozole treatment (Fig. 6), confirming that these take place along microtubules (Manneville et al., 2003).

We next analysed short-range WPB movements. WPB–actin interactions limit the movement of the organelle and can be analysed by determining the magnitude of short-range *x-y* displacements, the ‘cage radius’ (Manneville et al., 2003). The stronger the actin interaction, the more restricted WPB movements become hence reducing the cage radius. In agreement with the analysis of long-range movements, we found that, for WPBs in cells expressing MyRIP WT or MyRIP A751P, the cage radius was smaller than for cells expressing VWFpp–EGFP, whereas the cage radius was increased with MyRIP 4A or MyRIP A751P 4A (Fig. 5C).

### MyRIP–actin interaction prevents WPB exocytosis

**Fig. 7.**
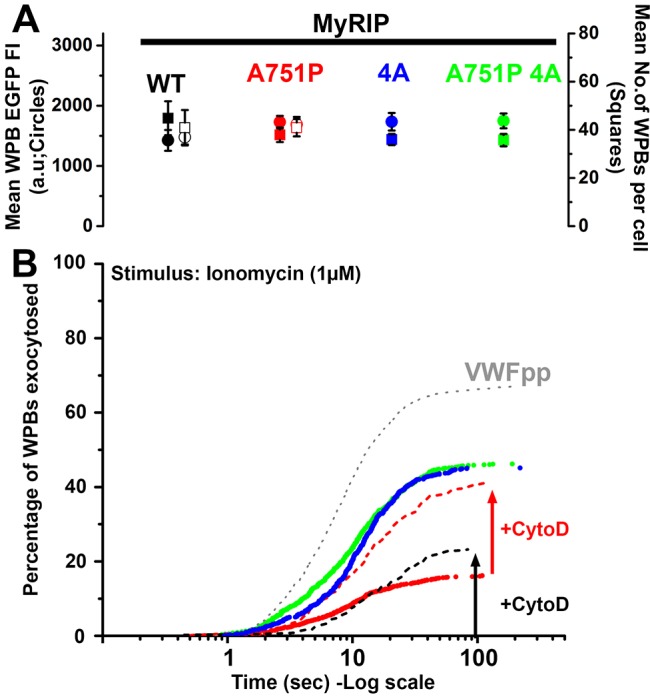
**MyRIP-actin interaction regulates WPB exocytosis.** (A) Mean±s.e.m. for the WPB-associated EGFP fluorescence intensity (solid circles) and numbers of fluorescent WPBs (solid squares) in cells expressing MyRIP WT (black, *n*=7), MyRIP A751P (red, *n*=28), MyRIP 4A (blue, *n*=23) and MyRIP A751P 4A (green, *n*=19). Open symbols show data for cells pre-treated with cytochalasin D (1 µM, 20 min). (B) Cumulative plots of the total number of WPB fusion events detected during ionomycin stimulation (1 µM, applied at *t*=0 s) for HUVECs expressing VWFpp–EGFP (grey dotted line, *n*=19 cells, 1074 fusion events, delay to first fusion event, 1.72±0.13 s; mean±s.e.m.), MyRIP WT (no trace, *n*=7, 0, 0 s), MyRIP A751P (red symbols, *n*=28, 219, 11.82±4.56 s), MyRIP 4A (blue symbols, *n*=23, 556, 5.05±0.97 s), MyRIP A751P 4A (green symbols, *n*=19, 557, 4.03±0.68 s). Dashed lines show data in cells treated with cytochalasin D (+CytoD, 1 µM, 20 min) expressing MyRIP WT (black dashed line, *n*=14, 91, 8.54±1.78 s) or MyRIP A751P (red dashed line, *n*=19, 320, 4.44±0.98 s). Delays were compared using ANOVA multiple comparisons with multiplicity adjusted; *P*-values are given in the main text. Data are scaled to the mean fraction of fluorescent WPBs that underwent fusion in each condition (VWFpp, 65.5±3.2%, *n*=19 cells; MyRIP WT, 0%, *n*=7 cells; cytochalasin-D-treated MyRIP WT, 23.2±1.7%, *n*=14 cells; MyRIP A751P, 17.6±3.4%, *n*=28 cells; cytochalasin-D-treated MyRIP A751P, 40.89±3.58%, *n*=19 cells; MyRIP 4A, 47.9±4.1%, *n*=23 cells; MyRIP A751P 4A, 50.1±4.1%, *n*=19 cells; mean±s.e.m.).

We next evaluated the effect of the EGFP–MyRIP mutants on Ca^2+^-driven WPB exocytosis. To directly compare the secretory responses between the different EGFP–MyRIP mutants, we selected cells that contained approximately equal numbers of fluorescent WPBs and which had similar WPB-associated EGFP fluorescence intensities (i.e. WPB EGFP–MyRIP mutant concentrations, see also Materials and Methods) (Fig. 7A). In this way, we aimed to minimise the effect on secretion of cell-to-cell variations in the amounts of each transgene on WPBs. Under these conditions, and consistent with previous findings (Bierings et al., 2012), expression of MyRIP WT completely inhibited WPB fusion (Fig. 7B). Expression of MyRIP A751P resulted instead, in a reduced inhibition of exocytosis, albeit with a slow onset compared to VWFpp–EGFP-expressing cells (delay 11.82±4.55 s, *n*=28 cells versus 1.72±0.13 s, *n*=19 cells, respectively, *P*=0.005, mean±s.e.m.) (Fig. 7B). Strikingly, with MyRIP 4A and MyRIP A751P 4A, WPB fusion events increased to ∼75% of those seen in VWFpp–EGFP-expressing cells (Fig. 7B), and the delays were not significantly different from those in cells expressing VWFpp–EGFP (5.05±0.97 s, *n*=23, *P*=0.75; 4.03±0.68 s, *n*=19, *P*=0.94, respectively). A recovery of WPB exocytosis to levels seen with VWFpp–EGFP was not expected because overexpression of MyRIP might displace other endogenous positive regulators of WPB exocytosis (e.g. Slp4-a or Munc13-4) that compete for binding to active Rab27A (Bierings et al., 2012; Zografou et al., 2012). To analyse further the contribution of the actin-binding property of MyRIP, we disrupted the actin cytoskeleton (cytochalasin D, 1 µM, 20 min) and determined the WPB secretory kinetics in cells expressing MyRIP WT and MyRIP A751P (Fig. 7B). Consistent with a prominent role of MyRIP–actin binding, we observed exocytosis of MyRIP-WT-expressing WPBs (delay 8.54±1.78 s, *n*=13), although the extent of exocytosis (29.3±4.0% of fluorescent WPBs, *n*=8) failed to reach that of MyRIP 4A (46±3.2% of fluorescent WPBs, *n*=23) (Fig. 7B). For MyRIP A751P, we found a significant decrease in the delay in exocytosis, to 4.14±1.26 s (*n*=9), that was now no different from that of WPBs expressing VWFpp–EGFP (*P*=0.99), MyRIP 4A (*P*=0.99) or MyRIP A751 4A (*P*=0.99). There was a significant increase in the extent of exocytosis from 16.4±2.4% (*n*=23) to 41.2±3.8% (*n*=19) of fluorescent WPBs (*P*=0.0001) (Fig. 7B), to a level now similar to that seen with cells expressing MyRIP 4A (*P*=0.90) or MyRIP A751P 4A (*P*=0.96). Finally, we determined the effect of actin disruption on Ca^2+^-driven VWFpp secretion in cells depleted of endogenous MyRIP by siRNA (Fig. S2C). Interestingly, although we did not observe a significant increase in VWFpp secretion upon MyRIP knockdown using ionomycin, we found a significant increase in VWFpp secretion in both cells treated with control siRNA siControl and siRNA against MyRIP (siMyRIP) upon cytochalasin D treatment. These results indicate that MyRIP regulates WPB exocytosis through its direct interaction with actin and MyoVa, and highlight the important point that other mechanisms involving the actin cytoskeleton must also play a role (see Discussion).

## DISCUSSION

The actin cytoskeleton plays multiple roles in the regulation of secretory granule trafficking and exocytosis (Porat-Shliom et al., 2013). Our previous work has shown that WPB–actin interactions regulate trafficking of this organelle close to the plasma membrane (Manneville et al., 2003), and here we have examined in more detail the molecular basis for this interaction.

Pharmacological disruption of the actin cytoskeleton produced a small, but statistically significant, increase in both WPB exocytosis and VWFpp secretion. These results are consistent with a model in which one of the roles of the actin cytoskeleton is to regulate Ca^2+^-stimulated WPB exocytosis. In addition, our new kinetic data suggest that upon treatment with cytochalasin D more WPBs became available for early release, contributing to the increased rate of WPBs fusing in the first 1–3 s. The physiological relevance of initially limiting the rate of exocytosis remains unclear, however, it might simply reflect the response time of the cellular machinery to the stimulus used. The failure of actin disruption to change the onset of exocytosis shows that there is a small pool of WPBs that have already overcome the actin hurdle and are ready to fuse, suggesting that the final steps in fusion are independent of actin. Consistent with this, failure of actin stabilisation to alter Ca^2+^-driven VWF secretion confirmed that acute actin re-modelling is not essential for this process.

Our findings agree with a previous study of hormone- and Ca^2+^-driven VWF secretion (Vischer et al., 2000), but differ in several respects from results obtained with the non-physiological secretagogue, phorbol myristate acetate (PMA) (Nightingale et al., 2009, 2011). During PMA stimulation, and in line with our observations, actin disruption results in an increase in the number of WPB fusion events. However, in contrast to our data and that of Vischer et al., 2000, PMA-induced VWF secretion is completely abolished (Nightingale et al., 2009). In the latter study, it was proposed that, in addition to acting as a barrier to WPB fusion, actin participates in the expulsion of VWF from WPBs through the formation and contraction of an actomyosin ring around the post-fusion WPB (Nightingale et al., 2011). However, recent analysis shows that this actomyosin mechanism does not contribute to Ca^2+^-driven WPB exocytosis and VWF secretion (Conte et al., 2015). We suggest that a main function of the actin cytoskeleton during Ca^2+^-driven exocytosis is to prevent rather than promote WPB exocytosis by restraining WPBs, limiting their access to the plasma membrane for fusion.

We next focused on the molecular components of the WPB that contribute to the interaction with actin. MyRIP has been described as a negative regulator of WPB exocytosis through its capacity to recruit MyoVa to clamp WPBs in the peripheral cortical actin, preventing exocytosis (Nightingale et al., 2009). However, studies in other cell types suggest that direct MyRIP–actin interaction might also play an important role in the regulation of secretory granule trafficking and exocytosis (Desnos et al., 2003; Huet et al., 2012; Waselle et al., 2003). The melanocyte Rab27A–MyoVa adapter protein melanophilin is closely related to MyRIP (Fukuda, 2008). Melanophilin binds MyoVa through several distinct regions including a MyoVa-globular-tail-binding domain (GTBD), a MyoVa-exon-F-binding domain (EFBD) and an amphipathic helix (MMAH) region towards the C-terminal end of the protein (Hume et al., 2006). The GTBD is not conserved in MyRIP, and mutagenesis studies have shown that only the MMAH region is essential for MyoVa binding by MyRIP, whereas other regions including the EFBD are non-essential for this interaction (Ramalho et al., 2009). Deletion or specific single point mutations within the MMAH effectively disrupt MyoVa binding and result in a failure of the mutant MyRIPs to rescue melanosome transport defects in melonophilin-null melanocytes (Ramalho et al., 2009). Because the actin-binding region of MyRIP is also located in the C-terminus of the protein (Kuroda et al., 2003; Waselle et al., 2003), a simple C-terminal truncation will disrupt both actin and MyoVa binding, complicating the analysis of the contributions of actin and/or MyoVa binding in MyRIP function. In this light, we chose to make a single point mutation in the MMAH of MyRIP that strongly disrupts MyoVa binding (Ramalho et al., 2009) while retaining the crucial actin-binding motif. Single-molecule imaging of the MyRIP mutants we generated showed that whereas the point mutation in the MMAH of MyRIP (MyRIP A751P) did not affect MyRIP binding to actin, the mutations on the actin-binding domain (MyRIP 4A) had a drastic effect, completely abolishing MyRIP–actin interactions. Taken together with the observation that mutations of the actin-binding domain did not perturb MyoVa recruitment to WPBs (Fig. 3F,G), our data strongly indicate that MyRIP binding to actin is independent of the ability of MyRIP to recruit MyoVa.

Our single-molecule dwell time analysis in live HUVECs showed that cytosolic MyRIP interacts weakly with actin, with a fast off rate (Fig. 4E) that could provide a mechanism to allow free MyRIP to diffuse and recycle to WPB-associated Rab27A. Indeed, increased diffusion, due to weak actin binding of free ATP-loaded MyoVa, has previously been suggested to be important for the efficient recycling of that motor protein to new cargo (Thirumurugan et al., 2006). Rab27A cycles on and off WPBs slowly (Bierings et al., 2012), a process thought to be regulated by the slow GTP-GDP exchange cycle for Rab27A (Kondo et al., 2006; Larijani et al., 2003). Our preliminary observations using whole WPB bleaching to study the timecourse for cycling of EGFP–MyRIP onto the WPB, suggest that once associated with endogenous (or exogenously expressed) Rab27A, MyRIP becomes tightly bound (Fig. S3). The slow cycling of MyRIP onto WPBs is no different from that of Rab27A itself (Bierings et al., 2012), suggesting that it is governed by the Rab27A GTP-GDP exchange cycle (Kondo et al., 2006; Larijani et al., 2003). The slow cycling of MyRIP is likely to account for its striking accumulation on WPBs, and, if the off rate for the MyRIP–actin interaction remains weak when bound to Rab27A, then concentrating many MyRIP molecules on the organelle will tend to favour an increase in the avidity of the MyRIP–actin interaction enhancing a tethering function. It is also possible that the strength of individual interactions between MyRIP and actin might increase when MyRIP is incorporated into its cargo-binding complex, as proposed for MyoVa (Thirumurugan et al., 2006), and for EGFP–Rab27A–melanophilin complexes bound to actin *in vitro* (Wu et al., 2006). Thus, weak actin binding in the free state will prevent MyRIP sequestration onto actin and allow it to accumulate on WPB–Rab27A and participate in clamping the granule into the actin cytoskeleton.

Analysis of the mobility of WPBs overexpressing EGFP–MyRIP WT revealed a drop in the proportion of WPBs with long trajectories, arguably caused by increased interaction with F-actin that counteracts WPB motions on microtubules (Manneville et al., 2003). This interpretation is supported by a complete abolition of long-range movements upon microtubule disruption (Fig. 6). This behaviour did not change for WPBs carrying MyRIP A751P and only when direct MyRIP–actin interactions were disrupted (MyRIP 4A) did we observe an increment in the proportion of WPBs with long-range movements. In this respect, the actin-binding-deficient mutant phenocopies the depletion of endogenous MyRIP. Consistent with our findings, an increased proportion of secretory granules with long trajectories has also been observed in neuroendocrine cells subjected to MyRIP silencing, together with an increment in secretory granule velocities (Huet et al., 2012)*.* Our kinetic analysis highlights the striking result that the role of MyRIP in WPB trafficking is not mediated by MyoVa, instead, MyRIP–actin interactions are the ones guiding the participation MyRIP in WPB mobility. However, a role for MyoVa–actin binding or transport in regulating WPB movements cannot be ruled out because MyoVa might potentially be recruited to WPBs through Rab27A–MyRIP-independent mechanisms (Lindsay et al., 2013; Wollert et al., 2011). In this context, other MyoVa partners might have a more prominent role in assisting the part that MyoVa plays in the movement of WPBs. Potential partners include Rab3 isoforms (Lindsay et al., 2013; Wollert et al., 2011), which are known to localise to WPBs (Bierings et al., 2012; Karniguian et al., 1993; Zografou et al., 2012). Moreover, a brain splice isoform of MyoVa has also been shown to bind both Slp4-a and rabphilin-3A in pancreatic β-cells (Brozzi et al., 2012a). Although this MyoVa isoform was not detected in HUVECs (Rojo Pulido et al., 2011), it only makes it more pressing to further our understanding on the complex mechanisms of MyoVa recruitment to WPBs.

Using high-speed live-cell TIRFM imaging, we analysed the effect of the expression of MyRIP mutants on Ca^2+^-driven WPB exocytosis. In the wild-type state, endogenous MyRIP has to compete for GTP-Rab27A with at least two other effectors, Slp4-a and Munc13-4 (Bierings et al., 2012; Zografou et al., 2012). Both of these effectors are positive regulators of WPB exocytosis, and Slp4-a in particular can also bind GDP-Rab27A, potentially allowing it to compete more effectively for Rab27A than MyRIP and Munc13-4 (Bierings et al., 2012). Competition between these different effectors balances the forces promoting and preventing exocytosis. In the wild-type state, the balance favours exocytosis (Bierings et al., 2012). Overexpression of MyRIP tilts the balance towards strong inhibition of secretion, through its ability to bind actin and MyoVa, and also potentially by displacement of the positive regulators Slp4-a and Munc13-4 from Rab27A. Being aware of these issues, we minimised cell-to-cell variations in WPB-associated MyRIP mutant concentrations by analysing cells with similar numbers of WPBs and with similar mean WPB EGFP fluorescence intensities (Fig. 7A,B). In this way, we hoped to compare more objectively the impact of a specific MyRIP mutation on WPB exocytosis kinetics. We found that specifically removing the ability of MyRIP to bind actin largely abolished its inhibitory effect, showing that direct actin binding underpins the negative function of MyRIP. Consistent with this, we found that disruption of the actin cytoskeleton in cells expressing MyRIP A751P reduced the delay and increased the extent of exocytosis to levels similar to those for MyRIP 4A and the combined mutant. Taken together, our results strongly support a model in which MyRIP–actin interactions play the dominant role in the function of MyRIP.

However, the contribution of the MyRIP–MyoVa interaction we observed in exocytosis (Fig. 7B), in contrast to trafficking (Fig. 5), should not be overlooked. Previous studies have shown that knockdown of MyoVa results in an increase in histamine-evoked VWF secretion (Rojo Pulido et al., 2011), and consistent with this we found that disruption of the MyoVa interaction with MyRIP, using MyRIP A751P, resulted in the appearance of WPB fusion events, albeit with a long delay and to a lesser extent compared to in cells expressing MyRIP 4A. This observation, coupled with the lack of effect of jasplakinolide on Ca^2+^-driven VWF secretion, suggest that there is a role for MyoVa in a later step in WPB exocytosis. Intriguingly, we found an increase in VWF secretion upon cytochalasin D treatment in MyRIP-depleted cells, suggesting that MyRIP–actin interactions are not solely responsible for preventing WPB exocytosis. It is interesting to speculate that MyoVa might, through alternative binding partners (see discussion above), provide some measure of functional redundancy in the regulation of WPB–actin interactions and exocytosis. Interestingly, MyoVa has been shown to play a positive role in exocytosis in other cell types. Under conditions of strong stimulation where [Ca^2+^]_i_ is elevated to high levels, calmodulin is known to dissociate from MyoVa IQ motifs (Sellers et al., 2008). In neurons, syntaxin-1A can bind to unoccupied MyoVa IQ motifs to promote assembly of the vesicle fusion apparatus (Watanabe et al., 2005) and, although this possibility has not been explored in endothelial cells, it is tempting to speculate that syntaxins 2, 3 or 4, which have been implicated in WPB exocytosis (Fu et al., 2005; van Breevoort et al., 2014), might also bind unoccupied MyoVa IQ motifs to modulate the function of the fusion apparatus.

In pancreatic β-cells, MyoVa recruitment by MyRIP only occurs after triggering PKA activation through the cAMP pathway, resulting in phosphorylation of rabphilin-3 (Brozzi et al., 2012b). Although, we could not detect any rabphilin-3 in HUVECs (data not shown), it shares a similar structure to Slp4-a, which is phosphorylated upon thrombin stimulation (van den Biggelaar et al., 2014), raising the intriguing possibility that MyoVa is recruited to WPBs in an activation-dependent fashion to regulate fusion. Thus, the function of MyoVa might not be as straightforward as initially imagined, depending on its mode of recruitment and activation in a space-and-time-appropriate manner.

Here, we have demonstrated that MyRIP directly mediates WPB interactions with the cortical actin network. This action does not rule out the proposed involvement of MyRIP in docking secretory granules to the plasma membrane (Huet et al., 2012) and the interaction of MyRIP with the exocyst complex (Goehring et al., 2007), which might not have been affected owing to the retention of the C-terminal domain, allowing the very pronounced recovery of exocytosis upon removal of the MyRIP–actin clamp. Our data suggest that trafficking and exocytosis of WPBs, which result from the complex interplay of a cocktail of Rab GTPases, effectors and other secretory mediators, can only begin to be understood by careful analysis of the molecular contributions of the individual components.

## MATERIALS AND METHODS

### Reagents

All reagents were from Sigma-Aldrich (Gillingham, UK) unless otherwise stated. Fura-2/AM and Rhodamine or FITC-conjugated phalloidin were from Life Technologies (Paisley, UK).

### Tissue culture, RNAi knockdown, ELISAs and immunocytochemistry

HUVECs were cultured and transfected as described previously (Erent et al., 2007). Endogenous MyRIP was depleted by RNAi using specific oligonucleotides (GE Healthcare, Little Chalfont, UK), and western blot analysis was carried out as previously described (Bierings et al., 2012). Biochemical secretion assays were carried out in release medium (serum-free M199, HEPES; 10 mM, pH 7.4) at 37°C, and secreted VWF and VWFpp were assayed by ELISA as described previously (Hewlett et al., 2011). Immunocytochemistry was performed as described previously (Bierings et al., 2012). Antibodies and dilutions used were as following: mouse monoclonal anti-GFP antibody (clone 7.1 and 13.1, 1:500) from Roche (Welwyn Garden City, UK), rabbit polyclonal anti-RFP antibody (ab62341, 1:200) and goat polyclonal anti-MyRIP antibody (ab10149, 1:200) from Abcam (Cambridge, UK), rabbit polyclonal anti-VWF antibody (A0082, 1:10,000) from Dako (Ely, UK), sheep polyclonal anti-VWF antibody (AHP062, 1:10,000) from Serotec (Kidlington, UK), rabbit polyclonal anti-VWFpp antibody (Babich et al., 2009; Hewlett et al., 2011), rabbit polyclonal anti-MyosinVa antibody (M4812, 1:100) from Sigma (Gillingham, UK). Fluorophore- or horseradish-peroxidase-coupled secondary antibodies were from Jackson ImmunoResearch Europe (Newmarket, UK).

### DNA constructs

VWFpp–EGFP, EGFP–MyRIP, EGFP–Rab27A and mRFP–Rab27A have been described previously (Bierings et al., 2012; Hannah et al., 2005; Kiskin et al., 2010). Flag–V159N-actin (Posern et al., 2004) was a kind gift from Guido Posern (MLU Institute for Physiological Chemistry, Halle, Germany). YFP–actin was from Clontech (catalogue number 6902-1). mCherry–actin WT and mCherry–actin V159N were made by introducing a XhoI site to the NcoI site at the N-terminus of the actin sequence using the following polylinker: CATGGCCTCGAGGC. Actin WT or and actin V159N were then excised using XhoI/XbaI and inserted into mCherry-C1. MyRIP mutants were generated by site-directed mutagenesis using EGFP–MyRIP (Bierings et al., 2012) as a template for generation of MyRIP A751P and MyRIP 4A and MyRIP 4A as a template for MyRIP A751 4A. Primers used had the following sequences: MYRIP A751P-f, 5′-GCCCAAGTCCACCATCCTGAACTCCAGATTTCAG-3′; MYRIP A751P-r, 5′-CTGAAATCTGGAGTTCAGGATGGTGGACTTGGGC-3′; MYRIP RftRRR-f, 5′-CATAGCACCATGTGTGGCCTTCACAGCAGCAGCGGATCAGAAGCAAAGG-3′; MYRIP RftRRR-r, 5′-CCTTTGCTTCTGATCCGCTGCTGCTGTGAAGGCCACACATGGTGCTATG-3′.

### Fluorescence microscopy

Confocal and wide-field fluorescence imaging were performed as described previously (Bierings et al., 2012). The kinetics and extent of WPB exocytosis in fura-2-loaded HUVECs expressing VWFpp–EGFP, EGFP–MyRIP or EGFP–MyRIP mutants was carried out using an Olympus IX71 inverted fluorescence microscope equipped with a Olympus UPLSAPO ×100 1.40 NA objective and enclosed within a microscope incubator at 37°C as previously described (Bierings et al., 2012; Kiskin et al., 2010). Data were acquired using the freeware software Winfluor (John Dempster, Strathclyde University; http://spider.science.strath.ac.uk/sipbs/showPage.php?page=software_imaging) and analysed as previously described (Erent et al., 2007). The probability of WPB exocytosis was determined as the mean fraction of fluorescent WPBs that underwent exocytosis following cell stimulation, as previously described (Hewlett et al., 2011). Confocal whole WPB bleaching and quantification of EGFP–MyRIP–WPB association kinetics was carried out as previously described (Bierings et al., 2012). For actin disruption, cells were treated with cytochalasin D (1 µM; 20 min). For comparison of the exocytosis kinetics between cells expressing different EGFP–MyRIP constructs, we selected cells with (1) similar numbers of fluorescent WPBs and (2) similar mean WPB EGFP fluorescence intensities. The mean WPB EGFP fluorescence intensity was determined in ImageJ from measurements of the EGFP fluorescence intensity of 10–20 individual WPBs in each cell from wide-field live cell time-lapse videos taken immediately prior to TIRF imaging of exocytosis. A user defined region of interest (ROI; polygon tool in ImageJ) was drawn around each WPB and the mean fluorescence intensity determined. The mean WPB EGFP fluorescence intensity for a cell was determined as the mean (±s.e.m.) of the individual WPB EGFP FI measurements made in that cell.

Single-molecule fluorescence imaging and WPB-tracking experiments were carried out using a custom-made total internal reflection fluorescence microscopy (TIRFM) setup. This set-up comprised a Zeiss Axiovert 135 inverted microscope (Carl Zeiss, Welwyn Garden City, UK) with an AlphaPlan, 100×, NA1.45 objective (Carl Zeiss), and an EMCCD camera (iXon-897BV, Andor, Belfast, UK) housed within a microscope incubator (Solent Scientific, Segensworth, UK) at 36°C (Hern et al., 2010). Laser excitation at 488 nm (100 mW) and 561 nm (150 mW) was provided by a LightHUB (Omicron, Germany). To minimise false detection events during automatic single fluorophore detection and analysis, cells expressing EGFP–MyRIP mutants containing few or no WPBs were selected for recording. Secretion, single-molecule or WPB-tracking data were acquired at between 10 and 50 frames/s (as indicated in the figure legends) using custom software and analysed using GMimPro/Motility freeware software (Gregory Mashanov, Francis Crick Institute Mill Hill Laboratory, London, UK, www.mashanov.uk). Estimates of the off rates for WT and mutant MyRIP from cortical actin or stress fibres were obtained from analysis of single EGFP fluorophore dwell times as previously described (Mashanov et al., 2004). The automatic single particle tracking (ASPT) module in GMimPro (Mashanov and Molloy, 2007) was used to track the *x*,*y* position in time of WPBs expressing VWFpp–EGFP, EGFP–MyRIP or EGFP–MyRIP mutants, yielding maximum velocities (using a seven-frame window) and maximum displacements for individual WPBs. ASPT settings used were FWHM500nm, R7, L100, Q10 and C3000. Data from multiple cells for each condition were automatically pooled using Motility software and time, *x* and *y* positions for WPBs were exported in text file format for subsequent analysis of mean squared displacement (MSD) in Matlab using custom-written functions. MDS plots were fitted by a nonlinear least squares to the equation MSD(*t*)=A+B×*t*^C^, where A is the offset, B is the diffusion coefficient (only correct for C=1), *t* is the time interval between frames and C is the exponent, the magnitude of which gives the general class of organelle diffusive behaviour; for free diffusion C=1±0.2, for subdiffusive or restricted diffusion C≤0.8, and for hyper diffusive or directed diffusion (C≥1.2). Individual data fits with an *r*^2^<0.8 were excluded from analysis. WPBs showing subdiffusive or restricted diffusion were further fitted using equation 3 from Manneville et al. (2003) to determine the cage radius and cage diffusion coefficient for restricted diffusion.

### Statistical analysis

Data were plotted in Origin 9.1 or GraphPad Prism 6.0. Statistical analysis was by *t*-test (non-parametric), or by one-way ANOVA using Tukey multiple comparisons test, as indicated, and was performed with GraphPad Prism 6.0. Significance values (*P*) for *t*-test, or multiplicity adjusted *P* values for ANOVA multiple comparisons are shown in the text, on the figures or in figure legends were appropriate. Data are shown as mean±s.e.m.
